# Long Term Metabolic Outcomes Following Pancreatectomy and Autologous Islet Transplantation: Systematic Review and Meta‐Analysis

**DOI:** 10.1002/jso.70193

**Published:** 2026-01-08

**Authors:** Daniel L. Hughes, Caterina Di Bella, Benedetta Quaratino, Pietro Rigo, Giulia Cirillo, Gioia Sgrinzato, Umberto Cillo, Lucrezia Furian, Giovanni Marchegiani

**Affiliations:** ^1^ Department of Hepato‐Pancreato‐Biliary Surgery Oxford University Hospitals NHS Foundation trust Oxford; ^2^ Department of Surgery, Oncology and Gastroenterology (DiSCOG) University of Padua Italy; ^3^ Diabetes Cell Therapy Center of the Veneto Region Padua Italy

**Keywords:** Autologous Islet Transplantation, Metabolic outcomes, Total Pancreatectomy, TPIAT, type 3c diabetes

## Abstract

This systematic review and meta‐analysis assessed long‐term outcomes following total pancreatectomy with islet autotransplantation (TPIAT). Seventeen studies including 1332 patients were analyzed. The pooled insulin independence rate was 34%, with higher rates for non‐chronic pancreatitis indications (68%) versus chronic pancreatitis (33%). TPIAT is effective in preserving endocrine function. Further studies are needed to validate outcomes across extended indications and to standardize reporting, incorporating metabolic markers and patient‐reported quality‐of‐life endpoints over long‐term follow‐up.

## 1 Introduction

Historically, Total Pancreatectomy (TP) was preferentially avoided as a surgical procedure due to the fear of the associated post operative diabetes. Type IIIc diabetes is a consequence to pancreatic parenchymal tissue loss, either as a sequelae to surgical resection or fibrotic gland destruction due to pancreatitis [1]. Type IIIc diabetes is not only life limiting but, in some circumstances, can be life threatening due to severe disruption of glucose homeostasis [2]. This condition is defined by frequent and unpredictable fluctuations between hyper and hypoglycemia, leading to a cumulative worsening of diabetes‐related end organ damage, morbidity and reduced quality of life for patients [2]. Moreover, the additional endocrine alterations of absolute glucagon and pancreatic polypeptide deficiencies predispose patients to severe hypoglycemic episodes [3, 4].

Islet AutoTransplantation (IAT) following TP serves as a mitigation strategy against type IIIc diabetes. Through auto transplantation of the patient's islets cell complexes, this provides the opportunity to regain endocrine function and glucose homeostasis without the need of exogenous insulin [5, 6]. IAT was initially described and intended for patients with Chronic Pancreatitis (CP) undergoing surgery [7]. As such, the majority of the published literature focuses on metabolic outcomes in this specific cohort of patients.

Over recent years, the indications for IAT have been expanded [8]. As per the Milan protocol, non‐pancreatitis indications for TPIAT now include high risk pancreatic stump, extended parenchymal resections for benign/borderline disease and for the management of severe complications following pancreatic surgery [9]. However, the metabolic outcomes for these patient cohorts are unknown. The aim of this systematic review and meta‐analysis is to determine the long‐term metabolic outcomes following TPIAT, also considering the new extended indications for surgery.

## 2 Materials and Methods

This systematic review and meta‐analysis of the current literature was performed in line with Preferred Reporting Items for Systematic Reviews and Meta‐Analyses (PRISMA) guidance [10]. The review was prospectively registered on PROSPERO (566031).

A comprehensive literature search was performed in Pubmed, EMBASE and Cochrane archives in order to identify all studies published in the last ten years that evaluated post‐operative metabolic outcomes TPIAT for all indications according to the Milan Protocol. The main search strategy consisted of islet, autologous, transplantation, autotransplantation. Boolean operators were used to expand the search. The references of selected articles were manually searched for additional relevant studies.

Studies were eligible for inclusion if they met the following criteria: (1) study population consisting of patients of any age who were treated with total, partial or completion pancreatectomy followed by IAT for all indications according to Milan Protocol; (2) studies that contained at least one major metabolic outcome indicator; (3) studies that implemented a well‐defined RCT, cohort or case‐control study design. According to the search strategy, a restriction was placed on the study period between 2014 and 2024. The most complete study was manually selected whenever multiple articles were published using the same study population.

Studies were excluded if (1) they were on animals; (2) they were editorials, letters to editors, comments about other articles, case reports, review articles; (3) the study population was less than 15 patients; (4) the follow‐up after surgery was less than 6 months; (5) they had insufficient data to describe metabolic outcomes or if the estimates were not extractable from graphics. Any non‐English article was excluded.

A standardized data collection form was used to extract the following information from each study: patient demographics, indication for TPIAT, predefined metabolic outcomes and the adverse glycemic event rate. Each included article was formally assessed for methodological quality and risk of bias using MINORS criteria for non‐randomized studies and ROB assessment for RCTs.

The primary outcome measure was determining the long‐term insulin independence rate following TPIAT. Secondary outcome measures included insulin independence rate according to indication for surgery, long term metabolic outcomes and the rate of severe hypoglycemic episodes. All statistical analysis was performed using R Foundation Statistical software (R 3.6.3). A meta‐analysis of outcomes was carried out using a random‐effects model incorporating the DerSimonian–Laird method. Data was visualized through the creation of forest plots. Heterogeneity was determined by calculating the I^2^ value. I^2^ value was considered to represent high ( > 75%), moderate (25–75%) or low degrees ( < 25%) of heterogeneity. A *p* value < 0.05 was considered statistically significant.

## 3 Results

**Figure 1:**
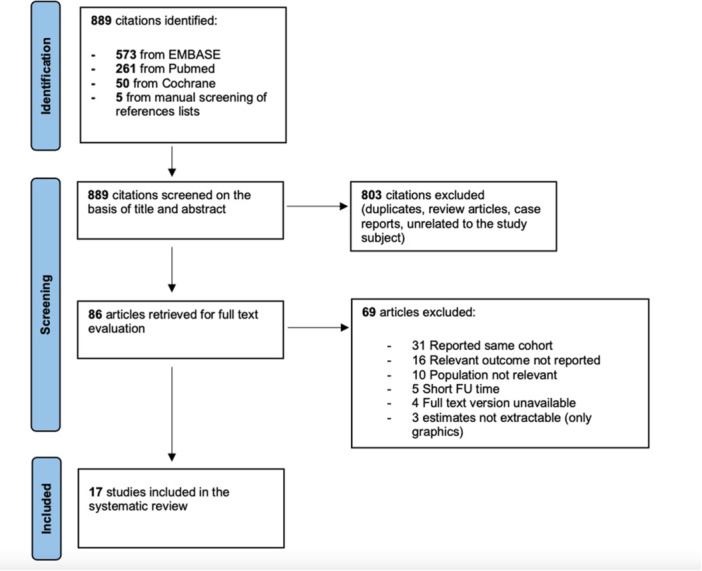
PRISMA diagram of included articles.

Following a systematic search through literature, a total of 889 articles were identified. After applying the predefined inclusion and exclusion criteria, 17 articles were included in the systematic review (Figure 1). During the screening phase, 31 articles were excluded because they were studies reporting the same cohort of patients. Upon study quality assessment, the median MINORS score was 14. All articles were considered of moderate quality (Supporting Information).

The 17 included articles comprised 1332 patients that had undergone TPIAT. The baseline demographics of the included patients are summarized in Table 1. The majority of the articles included (11/17, 65%) were conducted retrospectively. The individual sample size across all studies was small, as only 3 articles had a patient cohort > 100. Some 5 studies (29%) had recorded data on pediatric patients. The median follow‐up was 1 year.

**Table 1:** Descriptive demographics of the patients included by all studies.

Authors	Year of publication	Study design	Indication of IAT	FU since IAT	Study population, *n*	Age, mean (SD or range)/median (IQR or range)	Pediatrics
Darden et al [11]	2023	Retrospective	CP	1 y	157	39.5 (12.3)	N
Fröberg et al [12]	2023	Retrospective	CP, ARP Neoplasms	1 y	24	63 (23)	N
Lad et al [13]	2023	Prospective	CP	2.3 y	44	46 (20−78)	N
Pollard et al [14]	2023	Prospective	CP	5 y	60	44 (11)	N
Tellez et al [15]	2023	Retrospective	CP, ARP	1 y	40	13.1 (8.4−15.9)	Y
Vasu et al [16]	2023	Retrospective	CP	1 y	40	44 (32−48)	N
Chinnakotla et al [17]	2022	Retrospective	CP	4.1 y (1.56− 7.21)	564 (125 children)	34 (20−45), children 3‐17	Both
Ludwig et al [18]	2022	Retrospective	CP High risk stump Grade C POPF	3.8 y	24	57 (23)	N
Navas et al [19]	2022	Retrospective	CP, ARP	1 y	20	40 (10)	N
Swauger et al [20]	2022	Retrospective	CP, ARP	1 y	43	13.5 (8.9−16.5)	Y
Bampton et al [21]	2021	Prospective	CP	24 mo (14−45)	16	22 (15−36)	Both
Witkowski et al [22]	2021	RCT	CP, ARP	1 y	101	39.5 (12.2)	N
Bachul et al [23]	2020	Retrospective	CP	1 y	34	36 (15–51) DM group; 36 (16–52) pre‐DM group; 41 (9–60) non‐DM group.	Both
Quartuccio et al [24]	2017	Prospective	CP	1 y	34	39.3 (13.7)	N
Yoshimatsu et al [25]	2017	Retrospective	CP	1 y	54	40 (30.0−46.8)	N
Balzano et al [26]	2016	Prospective	High risk stump Neoplasms Grade C POPF	2.3 y 2 y 5 y	58	61 (14)	N
Yoon et al [27]	2015	Retrospective	Neoplasms	1 y	19	45.2 (16.5)	N

Abbreviations: CP= chronic pancreatitis; ARP= Acute Recurrent Pancreatitis; TP= Total Pancreatectomy; FU = Follow up

The indications for TPIAT were recorded (Table 1). Of the 17 selected studies, 13 papers involving a total number of 1101 patients focused exclusively on TPIAT for CP, while 2 papers involving a cohort of 67 patients considered indications other than CP for IAT. Specifically, the latter analyzed patients who underwent total or extensive pancreatectomy followed by IAT for neoplasms (26 patients), high‐risk anastomosis (28 patients), and POPF (13 patients). Additionally, 2 studies considered both patients undergoing IAT for CP and for other diseases. The study follow up duration was recorded. The length of follow up varied significantly between studied (range 1‐ 5 years). Only 4/17 (24%) of the studies included reported a follow‐up greater than 3 years.

Long‐term metabolic outcomes following TPIAT were recorded (Table 2). The individual insulin independent rate across all studies ranged from 0% to 50%. Partial graft function was recorded in 7 studies, with rates ranging from 2.9% to 71%. Graft failure was recorded in 7 studies, with rates ranging from 0% to 15%. Long term metabolic function at the end of the study follow up was also recorded.

**Table 2:** Metabolic outcomes following IAT.

Author	Islet infused IEQ/Kg^b^	Insulin independ. rate^f^	Partial graft function	Graft failure	Insulin req.^b^, ^e^	Fasting C‐pep.^b^	Stimulated C‐pep	Fasting glucose	Stimulated glucose	HbAc1(%)^b^ or % of patients on target	SHE^c^
Darden (2023)	5522 (2850)	33% (35/105)	ns	ns	12.4 ( ± 14.4) U/d	1.5 ( ± 1.3) ng/mL	ns	ns	ns	7.1 ( ± 1.9)	ns
Fröberg (2023)	2476 (947)	0% (0/24)	ns	ns	26.5 (±11.9) U/d	ns	ns	ns	ns	7.5 ( ± 3.5)	ns
Lad (2023)	7893 (4928−8936) *Independ*.	18.2% (8/44)	ns	ns	ns	ns	0.70 (0.60−0.70) ng/mL (peak)	ns	92.0 (86.0−100) mg/dL (peak)	5.9 (5.75−6.28)	ns
	4194 (3152−5484) *Dependent*						0.25 (0.20−0.43) ng/mL (peak)		138 (97.2−182) mg/dL (peak)	8 (7.68−9.43)	
Pollard (2023)	2166 (305−20,385)	29.4% (5/17)	ns	ns	0 U/d *Good responders* (*n* = 5)	ns	8.2 ( ± 4.7) ng/L	ns	8.4 ( ± 4.0) mmol/L	6.7 ( ± 1.4)	ns
					< 20 U/d *Partial responders* (*n* = 6)		3.2 ( ± 2.1) ng/L		24.9 ( ± 5.8) mmol/L	8.7 ( ± 1.8)	
					> 20 U/d *Poor responders* (*n* = 6)		1.5 ( ± 0.9) ng/L		25.5 ( ± 2.2) mmol/L	7.9 ( ± 1.9)	
Tellez (2023)	6328 (4298−8346)	32% (12/37)	ns	ns	ns	0.8 ng/mL (0.5−1.0), *n* = 14	1.8 ng/mL (1.2−2.2), (peak) *n* = 14	122.0 mg/dL (101.0−163.0), *n* = 14	ns	6.7 (5.9−7.8) MDI	ns
						0.7 ng/mL (0.5−0.8), *n* = 21	1.9 ng/mL (1.6−2.4), (peak) *n* = 21	97.0 mg/dL (87.0−110.0), *n* = 21		6.2 (5.6−7.4), *n* = 22, Pump	
Vasu (2023)	363,952^d^ (278,936−502,179)	45% (18/40)	ns	ns	0.10 (0.00−0.32) U/kg, *n* = 39	1.30 (0.62−2.00), ng/mL, *n* = 34	ns	108 (86−154), mg/dL, *n* = 37	ns	6.60 (5.93−7.47), *n* = 38	ns
Chinnakotla (2022)	3488 (2270.5− 5283.5)	35% (196/564)	49% (*n* = 429)	15% (*n* = 429)	ns	ns	ns	ns	ns	< 7^g^ Graft function group > 8^g^ Graft failure group	67 (12%)
Ludwig (2022)	3351 (676)	33% (8/24)	66% (16/24)	0%	0.09 ( ± 0.1) IU/kg/d	1.60 ( ± 0.88) ng/mL	ns	ns	ns	6.1 ( ± 1.0)	0
	2618 (2516)										
Navas (2022)	4294	45% (9/20)	30%	ns	ns	1.51 (0.1−3.7) ng/mL, *n* = 17	ns	ns	ns	7.7 (5.6−11.8), *n* = 18	2 (10%)
Swauger (2022)	9739 (6160−12,500) *Off insulin*	29% (12/41)	ns	ns	ns	ns	ns	ns	ns	ns	ns
	6120 (3992−6611) *Low ins. req*.	ns			< 0.5 U/kg/d *Low ins. req*. 51%						
	5044 (4212−7133) *High ins. req*.	ns			> 0.5 U/kg/d *High ins. req*. 20%						
Bampton (2021)	6650 (4660−10,500) *Independ*.	50% (8/15)	ns	ns	0.4 (0.15‐0.55) U/kg/d	ns	ns	ns	ns	5.3 (4.9−6.0)	
	3500 (1930−4240) *Dependent*									9.3 (9.0–10.0)	
Witkowski (2021)	4272 (2353)	20% (10/50)	ns	ns	0.17 ( ± 0.003) U/kg/d	ns	1.99 ( ± 0.18) ng/mL	ns	ns	6.4 ( ± 0.2)	5
	4049 (2406)	21% (11/51)			0.18 ( ± 0.003) U/kg/d		2.22 ( ± 0.19) ng/mL			6.8 ( ± 0.3)	0
Bachul (2020)	3400 (680−5190)	38% (11/29)	2.9% (1/34)	ns	ns	1.09 (0.30−1.96)ng/mL	ns	ns	ns	80% < 6.5	ns
	1297 (407−4190)					0.54 (0.09−1.57) ng/mL				37% < 6.5	
	2013 (34−2700)					0.24 (0−0.48) ng/mL				25% < 6.5	
Quartuccio (2017)	437,030^d^ (213,190− 775,970)	29% (10/34)	ns	ns	ns	ns	10 ( ± 2.2) ng/ml	ns	96 ( ± 7) mg/dL	5.5 ( ± 0.33) *Ins. Independ*.	ns
							8.8 ( ± 1.0) ng/ml		122 ( ± 9) mg/dL	8.0 ( ± 2.62) *Ins. dependent*	
Yoshimatsu (2017)	5320 (3657−7144)	45.3% (24/54)	ns	ns	0.11 (0−0.31) U/kg	0.95 (0.45−1.60) ng/mL	ns	ns	ns	6.8 (6.1−7.9)	ns
Balzano (2016)	2129 (1564−2665)	25% (7/28)	71% (20/28)	4% (1/28)	0.31 (0.12−0.53) U/kg/d	0.65 (0.2−1) ng/mL	ns	ns	ns	6.7 (6.1−7.5)	3
	1036 (594−1565)	94% (16/17)	6% (1/17)	0%	0.16 (0.09−0.63) U/kg/d	1.61 (1.31−1.87) ng/mL	ns	ns	ns	5.8 (5.3−6.1)	0
	1912 (1191−2476)	46% (6/13)	46% (6/13)	8% (1/13)	0.22 (0–0.47) U/kg/d	0.55 (0.3−1.24) ng/mL	ns	ns	ns	6.5 (5.85−8.05)	2
Yoon (2015)	1173 (839)	100% (9/9)	ns	ns	ns	ns	ns	ns	ns	5.9 ( ± 0.3)	ns

Abbreviations: ARP= Acute Recurrent Pancreatitis, CP= chronic pancreatitis, MDI= Multiple Daily Injections, ns= not specified, TP= Total Pancreatectomy.

* Indication of IAT is also classified according to Milano criteria (reported in brackets).

^a^ Months or years of FU since IAT. In some cases FU can be expressed as a median (range).

^b^ Data are shown as mean (SD) or median (range).

^c^ Severe Hypoglycemic Episodes, reported as number of patients who had ≥ 1 SHE (%).

^d^ Total islet equivalents (IEQ) is reported when islet infused IEQ/kg is not available.

^e^ Insulin requirement is shown in U/d when U/kg/d is not available.

^f^ Insulin independence rate achieved during follow‐up.

^g^ Mean values.

**Figure 2:**
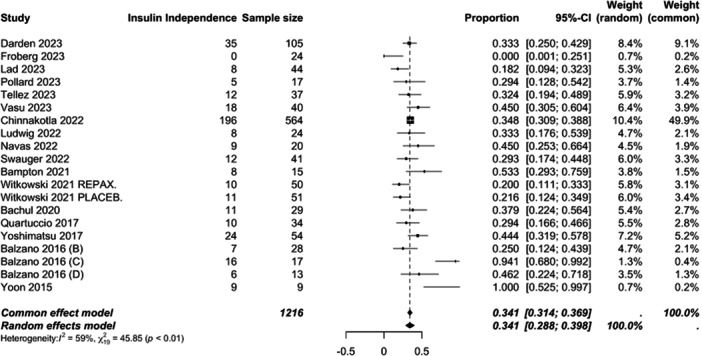
Forest plot of the overall pooled mean insulin independence rate.

In order to determine the overall rate of insulin independence across all studies, a meta‐analysis of pooled study data was performed (Figure 2). For insulin independence, across all 17 studies the pooled rate for insulin independence at the end of follow up was 34% (29−40%, moderate heterogeneity I^2^ = 59%). In order to further interrogate the data, additional analysis was performed by clustering patients as per the indications for TPIAT. In the context of TPIAT for CP, an overall insulin independent rate of 33% (29−38%, moderate heterogeneity I^2^ = 40%) was observed. However, a significantly higher insulin independent rate was observed for non‐pancreatitis TPIAT at 68% (27−93%, high heterogeneity ^I2^ = 83%).

**Figure 3:**
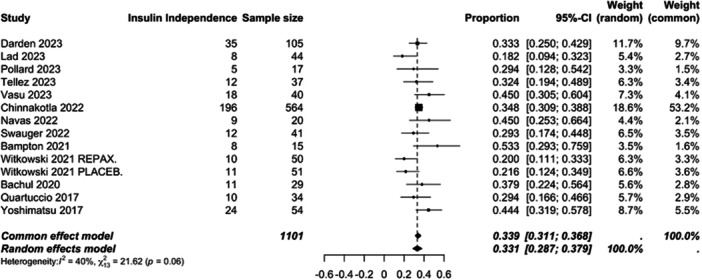
Forest plot of the pooled mean insulin independence rate calculated for IAT performed for CP.

Additional meta‐analyses of long‐term metabolic outcomes were also performed (supporting figure 1 and 2). The pooled mean HbA1c value following TPIAT was 6.9 (6.4–7.3, high heterogeneity I^2^ = 96%). The pooled mean fasting C‐peptide (ng/mL) value was 1 (0.76−1.25, high heterogeneity I^2^ = 93%). Where data was available a meta‐analysis of the pooled rate of severe hypoglycemic episodes was performed. The pooled event rate was 11% (9.2−14%, low heterogeneity I^2^ = 0%) Figure 3.

## 4 Discussion

This systematic review and meta‐analysis of the preexisting literature regarding TPIAT demonstrated that insulin independence (across all indications for TPIAT) was achieved in more than 30% of cases. As most patients are spared from developing type 3c diabetes and its associated complications, TPIAT proved to be a valid technique in terms of metabolic outcomes and should be available in centers of excellence for pancreatic surgery.

TPIAT remains a relatively novel technique. As a consequence, this is reflected within the literature with named institutions producing the majority of the published data. There is likely significant variation in islet isolation techniques and transplantation strategies [27]. This naturally introduces heterogeneity into the studies. This should be acknowledged when analyzing metabolic outcome data. Moreover, smaller centers or centers with a relatively new IAT program may be subject to the learning curve effect [28]. Suboptimal islet yield will impact long term endocrine outcomes and potentially introduce a bias into the data [28].

This systematic review illustrates that CP remains as the principal indication for TPIAT. Whilst other indications for TPIAT have been proposed, these were scarcely explored in the literature with only 4 of the 17 selected articles including patients undergoing TPIAT for non‐CP indications. As in particular the role of total pancreatectomy as a salvage strategy to avoid pancreatic fistula is extremely controversial in the literature [29, 30]. Another rare indication for TPIAT is in the context of pancreatic trauma, whereby resection of the injured gland (notably a distal pancreatectomy) will result in a significant reduction in pancreatic parenchymal mass and subsequently the patient will be at higher risk of developing type IIIc diabetes [31]. This is infrequently performed within clinical practice and is predominantly reported as single case reports within the literature [32, 33]. Further research is required for these cohorts of patients to define the role of IAT and to determine their long‐term outcomes.

An understandable concern amongst the surgical community regarding IAT in the management of malignant pancreatic disease is that of iatrogenic tumor seeding to the liver. Historically, malignancy was considered as an absolute contraindication to IAT [34]. In 2016, Muratore et al. published a case report (which to date remains the only one) on a case whereby a patient developed pancreatic cancer liver metastasis 10 months following IAT [35]. It should be noted that this patient underwent transplantation for CP rather than for malignancy. The authors proposed that additional screening adjunct such as microRNA may be of benefit [35]. Further research has been attempted to formally quantify the risk of tumor seeding. Dugani et al. published a murine based study whereby IAT was performed with islets procured from a genetic mouse model with a predisposition to pancreatic cancer and demonstrated no incidence of liver metastasis and no detrimental impact on survival [36].

Given recent advances in medical technology, the future of IAT may be contested. Current research efforts are focusing on developing artificial pancreas systems with continuous glucose monitoring. van Veldhuisen et al, published a randomized control trial comparing an artificial pancreas system and current standard of care diabetes management in patients undergoing total pancreatectomy [37]. They demonstrated that the use of the artificial pancreas system was associated with a significant increase in time spent in euglycemia in addition to a reduction in time in hypoglycemia [37]. In addition to being able to continuously monitor blood sugar, the artificial pancreas systems have the capability of infusing insulin and glucagon concurrently. As a consequence of having dual enzyme input, tighter physiological control can be achieved, and glucose homeostasis maintained.

Acknowledging the controversies about IAT for extended indications, this review demonstrates that the overall insulin independence rate of TPIAT is 34%. This finding is in keeping with other published studies [38]. Through expanding the analysis to the individual indications for IAT, a higher insulin independent rate is associated with non‐CP TPIAT (68% vs 33%). This is likely as a consequence to the fact that CP is associated with trans parenchymal fibrosis and gland destruction which ultimately diminishes the functional endocrine component, highlighting the importance of timely surgery for this indication. Although most studies in the literature focus on insulin independence rates, insulin independence should not be considered as the main outcome measure. Indeed, the main outcome should be twofold. Firstly, it should be the impact on the patients’ quality of life, notably by reducing time spent on hyperglycemia and preventing severe hypoglycemia episodes [39]. Through achieving this, not only does it cease end organ diabetes damage, but it also prevents recurrent hospital admissions for patients. Secondly, rather than focusing on insulin independence, diabetes control in general should be considered. This includes insulin requirements, degree of pharmacological supplementation required for euglycemia (c‐peptide as a proxy for graft function) and daily/long‐term glycemic control (glycated hemoglobin and episodes of severe hypoglycemia).

Several limitations were identified in this analysis. Since each center performing IAT relies on its own expertise and institutional protocols, marked variation in clinical practice occurs and significant heterogeneity is introduced into the results. Reporting of metabolic outcomes varied significantly across studies. No standardized reporting set of outcomes were noted. A consensus is required as to what defines successful outcomes in both terms of metabolic function and impact on quality of life. While safety and feasibility of IAT have been demonstrated for both CP and non‐CP indications, published data on metabolic efficacy remains predominantly centered around CP related IAT. With regard to the extended criteria for IAT, although preliminary results are promising, maturation of literature is required to understand the long‐term benefits. It should also be highlighted that most of the included studies were retrospective in nature and had a relatively short follow‐up. In the context of diabetes management, it is essential that studies report a longer follow up as only with time can one truly evaluate the impact of glycemic control on quality of life, and individuals’ ability to function socially and rates of end organ damage.

## 5 Conclusion

**Figure 4:**
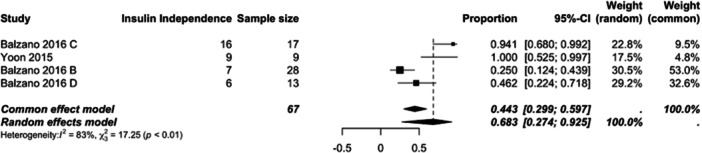
Forest plot of the pooled mean insulin independence rate calculated separately for IAT performed for indications other than CP (neoplasm, high risk anastomosis, grade C POPF).

IAT is a safe, reproducible, and effective technique for mitigating the risk of iatrogenic diabetes following pancreatectomy. Figure 4 This review demonstrates that an overall 34% insulin independence rate is achieved. A higher rate of insulin independence (68%) is achieved when IAT is performed for the extended criteria (non‐CP) indications for IAT. Further research is required to determine the long‐term metabolic outcomes for this patient cohort. The creation of a standardized outcome measure reporting set, incorporating metabolic function, oncological outcomes and quality of life data would allow for a comprehensive assessment of TPIAT given the rapidly expanding indications for its use.

## Funding

The authors received no specific funding for this work.

## Disclosure

The authors have nothing to disclose.

## Ethics Statement

The authors have nothing to report.

## Conflicts of Interest

The authors declare no conflicts of interest.

## Previous Publication

This data has not been previously published.

## Synopsis

This systematic review and meta‐analysis evaluated metabolic outcomes after TPIAT. Insulin independence was achieved in 34% of patients overall, and 68% of those undergoing surgery for non‐chronic pancreatitis indications. These findings support the expanded use of TPIAT and highlight the need for standardized outcome measures.

## Supporting information


**Supplementary figure 1.** Forest plot of the pooled mean value of glycated hemoglobin (%) following IAT. **Supplementary figure 2.** Forest plot of the pooled mean value of fating C‐peptide (ng/mL) following IAT. **Supplementary figure 3.** Forest plot of the pooled mean rate of severe hypoglycemia episodes following IAT. **Supplementary table 1.** Study quality assessment score (MINORS). **Supplementary table 2.** Risk of bias assessment of included RCTs. **Supplementary table 3.** Definitions of metabolic outcomes per study.

## Data Availability

Publicly accessible data.
