# Supplementary material for: Long Term Metabolic Outcomes Following Pancreatectomy and Autologous Islet Transplantation: Systematic Review and Meta‐Analysis
Source: J Surg Oncol. 2026 Jan 8;133(4):475–85. doi: 10.1002/jso.70193 (PMC12989187; doi:10.1002/jso.70193)
Supplement: Supplementary file 1 — Supplementary figure 1. Forest plot of the pooled mean value of glycated hemoglobin (%) following IAT. Supplementary figure 2. Forest plot of the pooled mean value of fating C‐peptide (ng/mL) following IAT. Supplementary figure 3. Forest plot of the pooled mean rate of severe hypoglycemia episodes following IAT. Supplementary table 1. Study quality assessment score (MINORS). Supplementary table 2. Risk of bias assessment of included RCTs. Supplementary table 3. Definitions of metabolic outcomes per study. [file JSO-133-475-s001.docx]

Supplementary figure 1

Forest plot of the pooled mean value of glycated hemoglobin (%) following IAT


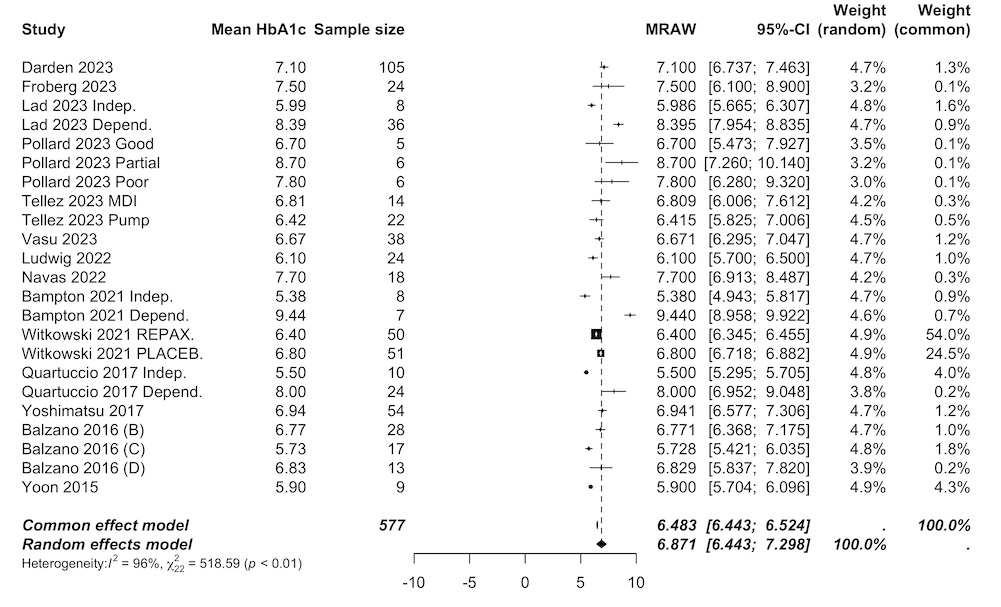


Supplementary figure 2

Forest plot of the pooled mean value of fating C-peptide (ng/mL) following IAT


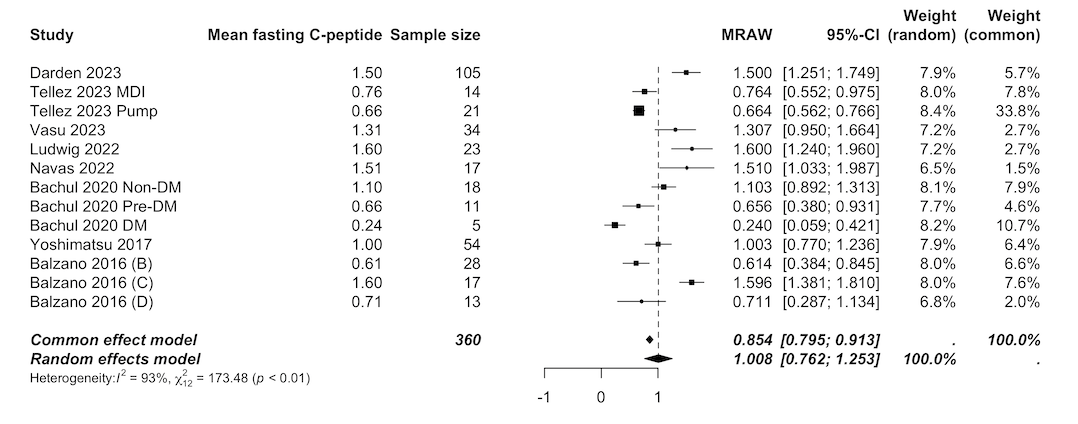


Supplementary figure 3

Forest plot of the pooled mean rate of severe hypoglycemia episodes following IAT


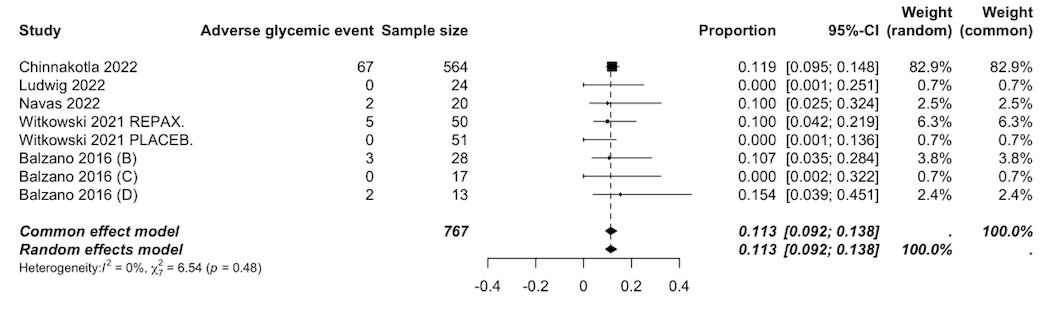


Supplementary table 1

Study quality assessment score (MINORS)

| Study | D1 | D2 | D3 | D4 | D5 | D6 | D7 | D8 | D9 | D10 | D11 | D12 | Total |
| --- | --- | --- | --- | --- | --- | --- | --- | --- | --- | --- | --- | --- | --- |
| Darden 2023 | 2 | 2 | 1 | 2 | 0 | 2 | 2 | 0 | 0 | 1 | 2 | 2 | 16 |
| Fröberg 2023 | 2 | 2 | 1 | 2 | 0 | 2 | 2 | 0 | 0 | 1 | 2 | 2 | 16 |
| Lad 2023 | 1 | 2 | 2 | 2 | 0 | 2 | 1 | 0 | 0 | 0 | 0 | 2 | 12 |
| Pollard 2023 | 2 | 2 | 0 | 2 | 0 | 2 | 1 | 0 | 0 | 0 | 0 | 2 | 11 |
| Tellez 2023 | 2 | 2 | 1 | 2 | 0 | 2 | 1 | 0 | 1 | 2 | 2 | 2 | 17 |
| Vasu 2023 | 2 | 2 | 1 | 2 | 0 | 2 | 1 | 0 | 0 | 0 | 0 | 2 | 12 |
| Chinnakotla 2022 | 2 | 2 | 1 | 2 | 0 | 2 | 2 | 0 | 0 | 0 | 0 | 2 | 13 |
| Ludwig 2022 | 2 | 2 | 1 | 2 | 0 | 2 | 1 | 0 | 0 | 0 | 0 | 2 | 12 |
| Navas 2022 | 2 | 2 | 1 | 2 | 0 | 2 | 2 | 0 | 2 | 1 | 2 | 2 | 18 |
| Swauger 2022 | 2 | 2 | 1 | 2 | 0 | 2 | 2 | 0 | 0 | 0 | 0 | 2 | 13 |
| Bampton 2021 | 2 | 2 | 1 | 2 | 0 | 2 | 1 | 0 | 0 | 0 | 0 | 1 | 11 |
| Bachul 2020 | 2 | 2 | 1 | 2 | 0 | 2 | 2 | 0 | 2 | 2 | 1 | 2 | 18 |
| Quartuccio 2017 | 1 | 2 | 1 | 2 | 0 | 2 | 2 | 0 | 0 | 0 | 0 | 2 | 12 |
| Yoshimatsu 2017 | 2 | 2 | 1 | 2 | 0 | 2 | 2 | 0 | 1 | 2 | 2 | 2 | 18 |
| Balzano 2016 | 2 | 2 | 2 | 2 | 0 | 2 | 2 | 0 | 0 | 0 | 0 | 2 | 14 |
| Yoon 2015 | 2 | 2 | 2 | 2 | 0 | 2 | 1 | 0 | 1 | 2 | 2 | 2 | 18 |

Domain 1- Clearly Stated Aim

Domain 2 - Inclusion of Consecutive Patients

Domain 3 - Prospective Data Collection

Domain 4 - Endppoints Appropriate to Study Aim

Domain 5 - Unbiases Assessment of Study Endpoint

Domain 6 - Follow-Up Period Appropriate to Study Aim

Domain 7 - <5% Lost to Follow-Up

Domain 8 - Prosective Calculation of Study Size

Domain 9 - An adequate control group

Domain 10 - Contemporary groups:

Domain 11 - Baseline equivalence of groups

Domain 12 - Adequate statistical analyses

Supplementary table 2

Risk of bias assessment of included RCTs

|  | Bias Domain | | | | | |
| --- | --- | --- | --- | --- | --- | --- |
| Study | Randomisation process | Deviations from intended interventions | Missing outcome data | Measurement of the outcome | Selection of the reported result | Overall bias |
| Witkowski 2021 | Low risk | Low risk | Some concerns | Low risk | Some concerns | Some concerns |

Supplementary table 3

Definitions of metabolic outcomes per study

| **Article** | **Insulin independence** | **Partial graft function** | **Graft failure** |
| --- | --- | --- | --- |
| Lad 2023 | No daily requirement of insulin and HbAc1<7% |  |  |
| Pollard 2023 | None | C-peptide > 0.3 ng/mL | C-peptide < 0.3 ng/mL |
| Vasu 2023 | None |  |  |
| Chinnakotla 2022 | None | Peak C-peptide ≥ 0.6 ng/mL (measured during MMTT) | Peak C-peptide < 0.6 ng/mL (measured during MMTT) |
| Ludwig 2022 | Off insulin while meeting glycemic targets (HbA1c <7%, fasting blood glucose levels <6.9mmol/l, and 2‐h postprandial blood glucose levels <10 mmol/l) | Fasting C peptide > 0.26 nmol/dL and need for exogenous insulin | Fasting C peptide <0.26nmol/dL |
| Navas 2022 | Off insulin and HbAc1< 6.5 | Requiring insulin but C-peptide > 0.5 | Fasting C-peptide ≤0.2 ng/mL |
| Swauger 2022 | Able to maintain HbAc1<7 for 14 days off insulin |  |  |
| Bachul 2020 | HbA1c <6.5% without the support of exogenous insulin or oral hypoglycemic agents |  | Undetectable fasting C-peptide on day 75 and afterward |
| Balzano 2016 | Off insulin with adequate glycemic control (HbAc1<7, fasting glucose <140 mg/dl >3 per week, 2-h post prandial levels <180 mg/dl >4 per week) | Fasting C peptide ≥ 0.2 ng/mL, need for exogenous insulin, or inadequate glycemic control (HbAc1>7, fasting glucose >140 mg/dl >3 per week, 2-h post prandial levels >180 mg/dl >4 per week) | Fasting C-peptide < 0.2 ng/mL |
